# Association of exacerbation phenotype with the sputum microbiome in chronic obstructive pulmonary disease patients during the clinically stable state

**DOI:** 10.1186/s12967-021-02788-4

**Published:** 2021-03-23

**Authors:** Chia-Yu Yang, Shiao-Wen Li, Chia-Yin Chin, Chia-Wei Hsu, Chi-Ching Lee, Yuan-Ming Yeh, Kuo-An Wu

**Affiliations:** 1 Department of Microbiology and Immunology, College of Medicine, Chang Gung University, Taoyuan, Taiwan; 2 Department of Otolaryngology-Head and Neck Surgery, Chang Gung Memorial Hospital, Taoyuan, Taiwan; 3 Molecular Medicine Research Center, Chang Gung University, Taoyuan, Taiwan; 4 Agricultural Biotechnology Research Center, Academia Sinica, Taipei, Taiwan; 5 Department and Graduate Institute of Computer Science and Information Engineering, Chang Gung University, Taoyuan, Taiwan; 6 Genomic Medicine Core Laboratory, Chang Gung Memorial Hospital, Linkou, Taiwan; 7 Department of Internal Medicine, Taoyuan Armed Forces General Hospital, No. 168, Zhongxing Rd., Longtan District, Taoyuan, 32551 Taiwan (R.O.C.); 8 School of Medicine, Fu Jen Catholic University, New Taipei City, 24205 Taiwan

**Keywords:** COPD, Sputum microbiome, Stable disease, Exacerbation risk, Lung function, 16S sequencing

## Abstract

**Background:**

Chronic obstructive pulmonary disease (COPD) is a progressive, life-threatening lung disease with increasing prevalence and incidence worldwide. Increasing evidence suggests that lung microbiomes might play a physiological role in acute exacerbations of COPD. The objective of this study was to characterize the association of the microbiota and exacerbation risk or airflow limitation in stable COPD patients.

**Methods:**

The sputum microbiota from 78 COPD outpatients during periods of clinical stability was investigated using 16S rRNA V3-V4 amplicon sequencing. The microbiome profiles were compared between patients with different risks of exacerbation, i.e., the low risk exacerbator (LRE) or high risk exacerbator (HRE) groups, and with different airflow limitation severity, i.e., mild to moderate (FEV1 ≥ 50; PFT I) or severe to very severe (FEV1 < 50; PFT II).

**Results:**

The bacterial diversity (Chao1 and observed OTUs) was significantly decreased in the HRE group compared to that in the LRE group. The top 3 dominant phyla in sputum were *Firmicutes*, *Actinobacteria*, and *Proteobacteria*, which were similar in the HRE and LRE groups. At the genus level, compared to that in the LRE group (41.24%), the proportion of *Streptococcus* was slightly decreased in the HRE group (28.68%) (p = 0.007). However, the bacterial diversity and the proportion of dominant bacteria at the phylum and genus levels were similar between the PFT I and PFT II groups. Furthermore, the relative abundances of *Gemella morbillorum*, *Prevotella histicola*, and *Streptococcus gordonii* were decreased in the HRE group compared to those in the LRE group according to linear discriminant analysis effect size (LEfSe). Microbiome network analysis suggested altered bacterial cooperative regulation in different exacerbation phenotypes. The proportions of *Proteobacteria* and *Neisseria* were negatively correlated with the FEV1/FVC value. According to functional prediction of sputum bacterial communities through Phylogenetic Investigation of Communities by Reconstruction of Unobserved States (PICRUSt) analysis, genes involved in lipopolysaccharide biosynthesis and energy metabolism were enriched in the HRE group.

**Conclusion:**

The present study revealed that the sputum microbiome changed in COPD patients with different risks of exacerbation. Additionally, the bacterial cooperative networks were altered in the HRE patients and may contribute to disease exacerbation. Our results provide evidence that sputum microbiome community dysbiosis is associated with different COPD phenotypes, and we hope that by understanding the lung microbiome, a potentially modifiable clinical factor, further targets for improved COPD therapies during the clinically stable state may be elucidated.

**Supplementary Information:**

The online version contains supplementary material available at 10.1186/s12967-021-02788-4.

## Background

Chronic obstructive pulmonary disease (COPD) is a common respiratory disease characterized by airflow limitation, lung function impairment, and airway inflammation. The incidence of COPD has increased worldwide, and it will become the third most prevalent cause of death by 2030 [1]. The Epidemiology and Impact of COPD (EPIC) Asia survey concluded that there was a high prevalence of COPD and a substantial socioeconomic burden of the disease in nine Asia–Pacific regions [2]. Airway obstruction, which is confirmed by spirometry, leads to air trapping and shortness of breath in response to physical exertion, and the poorly irreversible airway obstruction that characterizes COPD is progressive. In 2007, the grading of COPD severity proposed by the Global Initiative for Chronic Obstructive Lung Disease (GOLD) was based on forced expiratory volume in 1 s (FEV1) only. This classification could not adequately predict clinical outcomes [3, 4]. Exacerbations of COPD are important events in the disease course, and in particular, mortality in the year after an exacerbation requiring hospital admission is estimated to be as high as 21% [5]. The Evaluation of COPD Longitudinally to Identify Predictive Surrogate End-points (ECLIPSE) study suggested that individuals with two or more exacerbations in a given year represent a distinct frequent exacerbation phenotype [6]. However, exacerbations can occur across all stages of airflow limitation measured by FEV1, which emphasizes the need to identify other predictors of high exacerbation risk [6]. The GOLD 2011 revision presented an ABCD classification that combined respiratory symptoms, risks of exacerbations, and airflow limitations as indicated by FEV1 [7]. In 2017, The Global Initiative for Chronic Obstructive Lung Disease (GOLD) guide to COPD diagnosis and management used a threshold of two or more acute exacerbations in the previous year or at least one hospital admission related to an acute exacerbation to identify individuals at high risk of future events (groups C and D) and separated spirometric grades from the “ABCD” groupings [8].

Recent data have suggested that COPD is a complex and heterogeneous disease resulting from a number of different pathological processes, including infections [9]. Bacterial pathogens are commonly identified in the respiratory tracts of patients both in the stable state and during acute exacerbations, with significant changes in the prevalence of airway bacteria occurring during acute exacerbations of COPD [10–12]. An increased bacterial load has been associated with a decline in lung function [13] and increased rates of exacerbations in COPD patients [14], suggesting an important role of bacteria in the pathogenesis of COPD. Advances in next-generation sequencing platforms for 16S rRNA gene sequencing have provided opportunities to study the lung microbiomes in COPD patients, and the results have suggested that changes in the lung microbiota may be associated with enhanced airway inflammation and disease progression [15]. In patients with COPD, two large studies (AERIS study and COPDMAP study) have recently investigated the value of respiratory microbiome research to understand the association of microbiome changes and COPD exacerbations [16, 17]. Interestingly, both studies also identified alteration in the taxonomic composition of the lung microbiome related to the frequency of exacerbation. Pragman et al*.* reported that even during periods of clinical stability, the frequent exacerbation phenotype is associated with decreased alpha diversity, beta diversity clustering, and changes in taxonomic abundance [18].

Most patients with COPD are stable outpatients, and an important clinical challenge is to provide COPD outpatients appropriate education and prescribe appropriate therapy to prevent exacerbations. The current parameters for evaluating the severity of COPD patients are airflow limitation, symptoms, and exacerbation risks. Therefore, we undertook the present study to determine if the lung microbiome is associated with COPD clinical assessment parameters. Our hypothesis is that the lung microbiota alteration is important factor of exacerbation risk or airflow limitation in COPD outpatients during periods of clinical stability. We hope that by understanding the lung microbiome, a potentially modifiable clinical factor, further targets for improved COPD therapies may be elucidated.

## Methods

### Study subjects and study design

Seventy-eight subjects with COPD were enrolled in this study. The study was approved by the Institutional Review Board of the Tri-Service General Hospital Taiwan, and all subjects were enrolled from April 2015 to April 2016 and provided written informed consent. The patients were included if they were aged ≧ 40 years and had been diagnosed with COPD. We categorized each patient in our cohort according to GOLD 2017 classifications. Patients had no exacerbations or infections for at least 30 days prior to sample collection, and their sociodemographic and clinical data were recorded. For full inclusion and exclusion criteria see Additional file 1: Table S1. Symptoms were quantified with the modified Medical Research Council (mMRC) scale. The severity of exacerbations was classified as mild in the case of self-management with short-acting bronchodilators only; moderate if the patient was not hospitalized but received a prescription of systemic corticosteroids, antibiotics or both; and severe if the patient was hospitalized. The 78 COPD patients were classified by exacerbation risk as low-risk exacerbators (LREs, < 2 moderate exacerbations and no severe exacerbations per year, n = 60) and high-risk exacerbators (HREs, ≥ 2 moderate or severe exacerbations or ≥ 1 hospitalizations for COPD exacerbation, n = 18). Furthermore, according to pulmonary function testing, the 78 COPD patients were grouped as mild-to-moderate airflow limitation with a value of forced expiratory volume in the post-bronchodilator (post-BD) second FEV1% predicted ≥ 50 (PFT I, n = 43) and as severe-to-very severe airflow limitation with a value of post-BD FEV1% predicted < 50 (PFT II, n = 35).

### Sputum sampling and processing

Sputum was collected from patients by induction during stable visits after pulmonary function testing. All participants reported that they had no special dietary habits, had no known periodontal disease, had not taken systemic antibiotics in 4 weeks, and had not used antiseptic mouthwash before sample collection. Sputum was induced according to a previous protocol, with slight modifications [19]. For detailed methods for sputum sampling in this study, see Additional file 1.

### DNA extraction

Total bacterial genomic DNA was isolated from the sputum samples using the QIAamp DNA Microbiome Kit (Qiagen, USA). Briefly, 250 µl of AHL buffer was added to 500 µl of sample for host cell lysis, followed by digestion of the host nucleic acids with 1.25 μl of benzonase and 10 μl of proteinase K. The host DNA was separated by centrifugation, after which 100 μl of ATL buffer was added to the bacterial cells in a pathogen lysis tube L and the sample was vortexed using a TissueLyser LT for 10 min at 30 Hz. The bacterial DNA was washed, eluted using nuclease-free water, and stored at − 80 °C. The concentrations and qualities of the purified DNA were determined with a Qubit high-sensitivity dsDNA assay (Life Technologies).

### Sputum microbiota profiling by 16S rRNA gene sequencing

A 16S rRNA gene amplicon library targeting the 16S rRNA V3-V4 region was constructed as in a previous report [20]. Illumina adaptor overhang nucleotide sequences were added to the gene-specific forward and reverse primers. Two-round PCRs were performed, and the final amplicon libraries were approximately 630 bp in length. The multiplex amplified libraries were pooled equally and sequenced on a MiSeq system with 2 × 300 paired-end v3 sequencing reagents (Illumina, USA). For detailed methods for 16S rRNA gene sequencing, see Additional file 1.

### Bioinformatics analysis

The sequencing reads were processed, and the taxonomic classification was performed using FLASH (version 1.2.11) [21]. Low-quality reads were filtered [22], and only sequence tags with lengths > 400 bp were retained for subsequent analysis. The operational taxonomic units (OTUs) were clustered at 97% sequence similarity using USEARCH (version 9.2.64) [23] against the Greengenes 16S rRNA gene database (13_8 release), and final taxonomic assignments were performed using the RDP classifier [24]. Furthermore, our results were validated by another bioinformatic pipeline using the DADA2 package [25] for modelling and amplicon error correction, which was followed by quality filtering, dereplication, denoising, merging and chimaera removal. A naïve Bayes classifier [24] was trained using the most recent available version of the Silva (version 132) sequences for taxonomic assignments. A bivariate correlation analysis of the 15 most abundant genera using Spearman’s correlation coefficient was performed in R. We then constructed a co-occurrence network of the predominant sputum microbiota with different COPD disease severities. The network was generated using Cytoscape (version 3.7.0) and visualized using a circular layout [26]. Potential biomarkers were determined using linear discriminate analysis effect size (LEfSe) [27]. Microbial functionality profiles were predicted using Phylogenetic Investigation of Communities by Reconstruction of Unobserved States (PICRUSt) to generate the Kyoto Encyclopedia of Genes and Genomes (KEGG) pathways [28]. For detailed methods for bioinformatics analysis, see Additional file 1.

### Statistical analyses

Box and whiskers plots (10–90 percentile) of alpha diversity indices and taxonomic abundances comparing two groups were plotted using GraphPad Prism 6 (GraphPad Software, Inc., La Jolla, CA, USA). Principal component analysis plots for LRE vs HRE, PFT I vs PFT II, and smoker vs non-smoker were prepared using unweighted UniFrac distance. The Mann–Whitney Wilcoxon test followed by Bonferroni correction was used to test for significant differences in alpha diversity or taxonomic levels between groups. Bonferroni-adjusted p values were calculated as an alpha error of 0.05 divided by the numbers of parameters in each table. Spearman's correlation was used to determine the association of the dominant phyla or genera with FEV1/FVC, and p values were adjusted by using Bonferroni correction for multiple tests. Statistical analyses were performed by using R or SPSS (SPSS Inc., Chicago, IL, USA).

## Results

### COPD patient demographics and sputum microbiota profiling

To characterize the lung microbiome constituents that differentiate the low risk vs high risk exacerbation groups or mild-to-moderate vs severe-to-very severe airway limitation groups under stable COPD conditions, sputum specimens were collected from 78 COPD patients during stable visits. The characteristics of the patients are summarized in Table 1, and the detailed information regarding inclusion and exclusion criteria are available in Additional file 1: Table S1. The patients’ age range was 40–93 years, and none of the patients had been on antimicrobial therapy for the last 4 weeks prior to sample collection. Ages and smoking histories were similar in COPD patients at different stages. Among these 78 patients, 31 patients (39.7%) with COPD received a long-acting bronchodilator (LAB) and 45 patients (57.6%) received both long-acting β2 agonists (LABA) and inhaled corticosteroid (ICS) treatment. The 78 COPD patients were divided into two subgroups, including LRE (n = 60) and HRE (n = 18). Furthermore, the 78 COPD patients were grouped as mild-to-moderate airflow limitation with a value of FEV1% predicted ≥ 50 (PFT I, n = 43) and as severe-to-very severe airflow limitation with a value of FEV1% predicted < 50 (PFT II, n = 35).

**Table 1:** Clinical characteristics of the study population (N = 78) in this study

Characteristics	All	Low risk exacerbator (LRE)	High risk exacerbator (HRE)	PFT I	PFT II
Number of patients	78	60	18	43	35
Age (years)					
Range	40–93	40–91	52–93	40–90	52–93
Mean ± s.e.m	74.17 ± 1.446	73.63 ± 1.673	75.94 ± 2.889	72.37 ± 2.020	76.37 ± 2.025
Gender					
Male	68 (87.2%)	50 (83.3%)	18 (100%)	36 (83.7%)	32 (91.4%)
Female	10 (12.8%)	10 (16.7%)	0 (0%)	7 (16.3%)	3 (8.6%)
FEV1 (% predicted)					
Range	24.6–97.9	30.5–97.9	24.6–73.8	50.0–97.9	24.6–49.6
Mean ± s.e.m	53.24 ± 1.682	55.73 ± 1.902	44.95 ± 2.901	63.62 ± 1.702	40.49 ± 1.097
FEV1/FVC					
Range	37.31–69.54	45.33–69.54	37.31–66.86	52.30–69.54	37.31–69.18
Mean ± s.e.m	59.59 ± 0.767	60.98 ± 0.783	54.95 ± 1.673	62.45 ± 0.738	56.07 ± 1.218
Current smoking					
No	17 (21.8%)	13 (21.7%)	4 (22.2%)	11 (25.6%)	6 (17.1%)
Yes	61 (78.2%)	47 (78.3%)	14 (77.8%)	32 (74.4%)	29 (82.9%)
Medication					
LAB	31 (39.7%)	28 (46.7%)	3 (16.7%)	28 (65.1%)	3 (8.6%)
LABA + ICS	45 (57.7%)	30 (50.0%)	15 (83.3%)	13 (30.2%)	32 (91.4%)
No	2 (2.6%)	2 (3.3%)	0 (0%)	2 (4.7%)	0 (0%)

*LRE* low risk exacerbator, *HRE* high risk exacerbator, *PFT* pulmonary function test, *PFT I* FEV1 ≥ 50, *PFT II* FEV1 < 50, *FEV1* forced expiratory volume in the first second, *FVC* forced vital capacity, *LAB* long-acting bronchodilator, *LABA* long-acting β2 agonists, *ICS* inhaled corticosteroid

DNA isolated from the sputum specimens was subjected to two-round PCR amplification and library construction of the 16S V3-V4 region and was sequenced on an Illumina MiSeq system. The average number of raw reads was 228,741 for all COPD patients (Additional file 1: Table S2). After selecting the qualified reads, the average number of paired quality-filtered reads was 133,386 for all COPD patients (Additional file 1: Table S2). The rarefaction curve showed that the sequencing depth per sample was enough to represent most of the community diversity and reached a saturated plateau phase (data not shown).

**Fig. 1:**
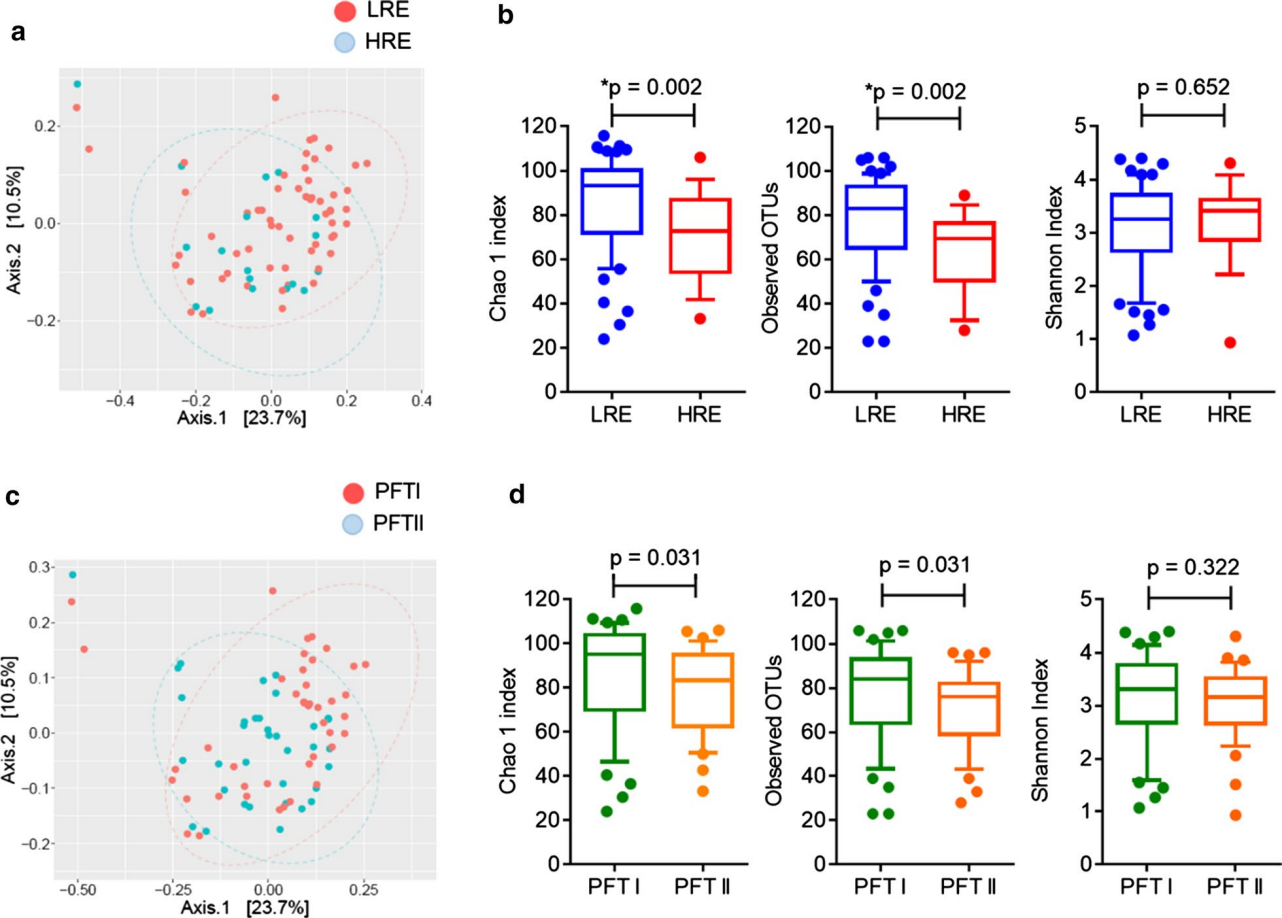
Analysis of the sputum microbiome and diversity in stable COPD. The unweighted PCA plot (**a**) and Chao1 index, observed OTUs, and Shannon index (**b**) were calculated in the LRE vs HRE groups. The unweighted PCA plot (**c**) and the diversity index (**d**) were presented in the PFT I vs PFT II groups. The box and whiskers plots show the median, 10th and 90th percentile in each group. Bonferroni-adjusted p-values < 0.05/6 = 0.0083 indicate significance. *LRE* low-risk exacerbator, *HRE* high-risk exacerbator

We first compared the bacterial compositions and diversity of the sputum microbiomes in different COPD phenotypic subgroups. Regardless of exacerbation phenotype, the unweighted principal component analysis (PCA) plot revealed a similar sputum microbiota between the LRE and HRE groups (Fig. 1a). Species richness according to Chao1 index (p = 0.002) and observed OTUs (p = 0.002) was lower in the HRE group, indicating that the sputum microbiota in these patients was characterized by a lower diversity than that in the LRE patients (Fig. 1b). The species evenness according to Shannon index was similar in the LRE and HRE groups (p = 0.652) (Fig. 1b). We then analysed the microbiome diversity in patients with different airway limitation severity. The unweighted PCA plot (Fig. 1c) and alpha diversity according to Chao1, observed OTUs, and Shannon indices (Fig. 1d) were similar between the PFT I vs PFT II groups. The bacterial compositions and diversity of the sputum microbiomes in smokers and non-smokers were similar (Additional file 1: Figure S1). These results indicated that the sputum microbiome was altered and that these compositional changes may have been associated with exacerbation risk.

### Most abundant bacterial taxa changes in stable COPD patients with high risk of exacerbation

**Fig. 2:**
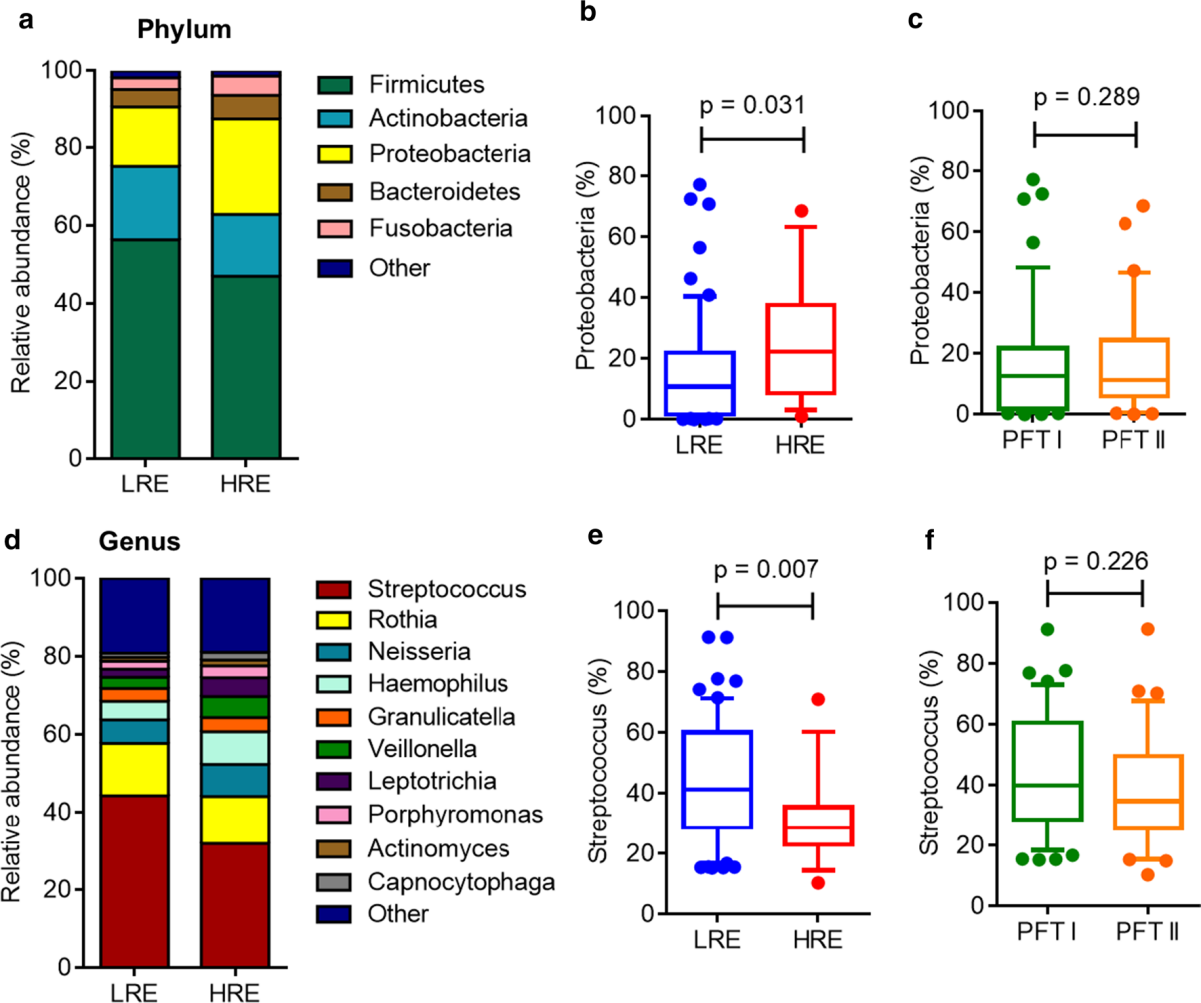
Relative abundance of the most prevalent bacteria at the phylum and genus levels in stable COPD patients. The bacterial taxonomic distributions are shown at the phylum and genus levels in the sputum microbiomes of COPD patients. The stacked bar represents differentially expressed bacteria at the phylum level (**a**) in different COPD phenotypic subgroups. The box and whisker plots (10–90 percentile) show the relative abundance of *Proteobacteria* in the LRE vs HRE groups (**b**) or in the PFT I and PFT II groups (**c**). The stacked bar represents differentially expressed bacteria at the genus level (**d**) in the different COPD phenotypic subgroups. The box and whisker plots show the relative abundance of *Streptococcus* in the LRE vs HRE groups (**e**) or in the PFT I and PFT II groups (**f**). The p value was analyzed using the Mann–Whitney Wilcoxon test. Bonferroni-adjusted p-values < 0.05/15 (5 phyla and 10 genera) = 0.0033 indicate significance. *LRE* low-risk exacerbator, *HRE* high-risk exacerbator

Figure 2 shows the dominant taxa at the phylum and genus levels. The sputum microbiome dataset from our cohort revealed a total of five phyla, which accounted for 99% of all the bacteria (Fig. 2a; Table 2). The top five dominant phyla were *Firmicutes*, *Actinobacteria*, *Proteobacteria*, *Bacteroidetes*, and *Fusobacteria*. *Firmicutes* was the most dominant phylum in stable COPD patients, with a relative abundance of 43.5–54.6%, with *Actinobacteria* being the second most dominant phylum, with a relative abundance of 16.6–16.7% (Table 2). The proportions of the dominant phyla were similar in the LRE vs HRE or PFT I vs PFT II groups (Fig. 2b, c; Table 2). The relative abundance of *Proteobacteria* was slightly increased but not significantly different in the HRE group compared with that in the LRE group (p = 0.031) (Fig. 2b).

**Table 2:** Taxonomic identification at the phylum and genus levels in COPD

Phylum	LRE	HRE	p value	PFT I	PFT II	p value
Patients	60	18		43	35	
Firmicutes	54.59 (41.54–73.32)	43.50 (31.63–60.68)	0.075	48.32 (40–66.09)	57.67 (38.70–69.2)	0.605
Actinobacteria	16.63 (8.48–28.24)	16.71 (11.58–22.79)	0.652	17.08 (8.62–27.99)	15.19 (10.11–24.00)	0.591
Proteobacteria	10.87 (1.69–22.06)	22.28 (8.70–37.70)	0.031	12.66 (1.90–21.82)	11.25 (5.96–24.55)	0.289
Bacteroidetes	3.26 (0.87–6.47)	3.98 (0.27–12.59)	0.610	3.51 (1.22–7.41)	3.18 (0.58–7.55)	0.706
Fusobacteria	2.00 (0.88–4.57)	2.95 (0.57–4.73)	0.585	2.36 (1.04–4.95)	1.65 (0.84–3.44)	0.083
Genera						
Streptococcus	41.24 (28.72–60.15)	28.68 (22.91–35.54)	0.007	39.94 (28.61–60.65)	34.83 (25.70–49.55)	0.226
Rothia	10.83 (4.31–18.67)	10.20 (4.82–21.14)	0.859	10.86 (4.50–19.53)	10.51 (3.96–16.65)	0.543
Neisseria	3.07 (0.34–10.51)	3.42 (0.82–11.06)	0.400	3.04 (0.39–9.95)	4.21 (0.31–13.13)	0.393
Haemophilus	2.72 (0.19–6.43)	1.34 (0.22–11.76)	0.962	3.21 (0.23–6.41)	2.38 (0.16–11.69)	0.868
Granulicatella	2.54 (1.57–3.93)	2.95 (1.71–5.54)	0.343	2.54 (1.33–3.64)	2.86 (1.77–4.39)	0.317
Veillonella	1.45 (0.73–3.19)	2.25 (0.69–5.25)	0.226	1.73 (0.71–4.11)	1.64 (0.89–3.48)	0.956
Leptotrichia	1.30 (0.42–2.67)	1.14 (0.32–3.62)	0.840	1.67 (0.49–3.51)	0.96 (0.36–2.60)	0.353
Porphyromonas	0.96 (0.03–3.29)	0.94 (0.04–4.50)	0.677	1.03 (0.05–3.39)	0.78 (0.02–3.02)	0.790
Actinomyces	0.55 (0.19–1.35)	0.92 (0.34–1.48)	0.215	0.82 (0.19–1.43)	0.50 (0.25–1.26)	0.802
Capnocytophaga	0.37 (0.06–1.58)	0.61 (0.08–1.88)	0.585	0.46 (0.09–1.73)	0.42 (0.04–1.19)	0.457

Data are presented as median (25–75 percentile)

*LRE* low risk exacerbator, *HRE* high risk exacerbator, *PFT* pulmonary function test, *PFT I* FEV1 ≥ 50, *PFT II* FEV1 < 50.0

The p value is analyzed using Mann Whitney Wilcoxon test. Bonferroni-adjusted p-values < 0.05/15 (5 phylum and 10 genus) = 0.0033 indicate significance

The top 10 dominant genera in sputum were *Streptococcus*, *Rothia*, *Haemophilus*, *Neisseria*, *Veillonella*, *Granulicatella*, *Porphyromonas*, *Leptotrichia*, *Actinomyces*, and *Capnocytophaga* (Fig. 2d; Table 2)*.* Among the top 10 genera, the relative abundance of *Streptococcus* was slightly decreased but not significantly different in the HRE group (28.68%) compared to that in the LRE group (41.24%) (p = 0.007) (Fig. 2e; Table 2). The relative abundance of *Streptococcus* was similar between the PFT I (39.94%) and PFT II groups (34.83%) (p = 0.226) (Fig. 2f). We also classified the participants into older-aged adults (60 years and above, n = 67). Among the older-aged COPD patients, the proportions of the dominant phyla and genera were similar in the LRE (n = 51) vs HRE groups (n = 16) (Additional file 1: Table S3). A trend of slightly increased *Proteobacteria* and decreased *Streptococcus* abundances was also observed in older-aged COPD patients with different risks of exacerbation. However, the taxonomic distribution was similar in smoker (n = 61) vs non-smoker (n = 17) COPD patients (Additional file 1: Table S4).

### Differential taxa in the microbiome of stable COPD patients

A LEfSe analysis was performed to identify the differences in taxonomic distributions associated with different COPD phenotypic subgroups. The cladogram plotted based on LEfSe analysis shows five taxonomic levels, with the phyla levels and genera levels plotted in the innermost ring and outermost ring, respectively (Additional file 1: Figure S2). The amount of *Pseudomonadales*, which is an order of the phylum *Proteobacteria*, was enriched and had the highest linear discriminant analysis (LDA) score in HRE subjects. Otherwise, the amounts of *Bacilli* and *Lactobacillales*, which is a subclass of phylum *Firmicutes*, were enriched with in LRE subjects (Additional file 1: Figure S2).

**Fig. 3:**
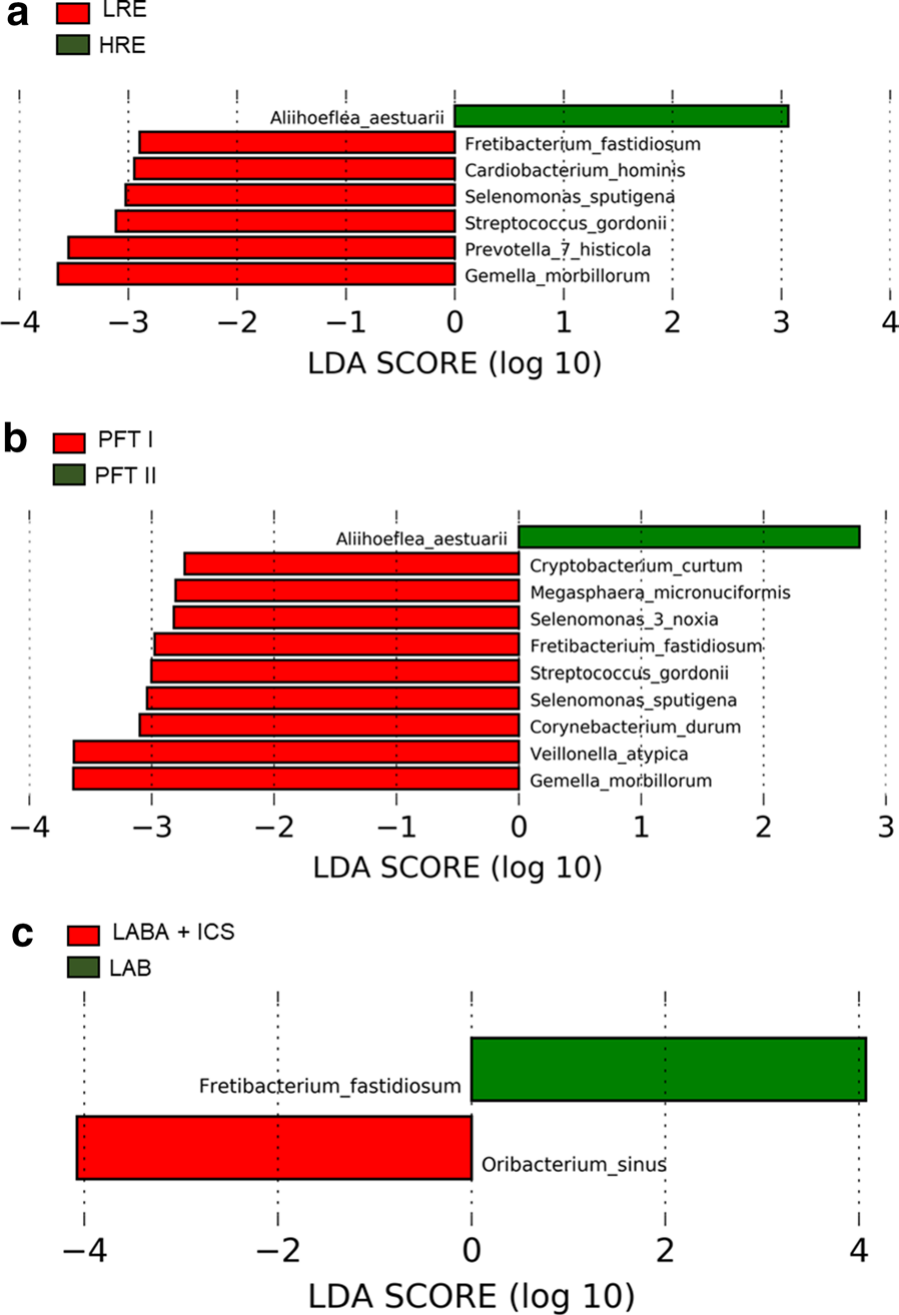
Linear discriminant analysis (LDA) effect size (LEfSe) revealed differentially abundant bacterial taxa in stable COPD. The plots from LEfSe analysis indicated differentially enriched bacteria in the LRE vs HRE groups (**a**), PFT I vs PFT II groups (**b**), and different medical treatments (**c**). *LRE* low-risk exacerbator, *HRE* high-risk exacerbator

To identify the differential bacterial taxa at the species level, we performed LEfSe analysis based on the known OTUs at the species level. Many bacteria at the species level were enriched in different subgroups of COPD; therefore, we selected a LDA score higher than 3 or lower than -3 to represent the most significantly enriched species in each group (Fig. 3). The relative abundances of *Gemella morbillorum* (*G. morbillorum)*, *Prevotella histicola (P. histicola)*, and *Streptococcus gordonii* (*S. gordonii*) were significantly decreased in HRE compared to LRE subjects (Fig. 3a). Furthermore, the relative abundances of *Gemella morbillorum* (*G. morbillorum*), *Veillonella atypica* (*V. atypica*), and *Corynebacterium durum* (*C. durum*) were significantly decreased in patients with severe-to-very severe airflow limitation compared with patients with mild-to-moderate airflow limitation (Fig. 3b). In our cohort, the percentages of patients who were treated with a long-acting bronchodilator (LAB) or long-acting β2 agonists (LABA) and an inhaled corticosteroid (ICS) were approximately 39.7% and 57.7%, respectively. We also compared the microbiomes of COPD patients who received different medications. The Chao1 index and observed OTUs were similar between these two medications (Additional file 1: Figure S3). The proportions of *Fretibacterium fastidiosum* and *Oribacterium sinus* were enriched in LAB and LABA plus ICS, respectively (Fig. 3c).

### Correlation between lung function and the microbiome

**Fig. 4:**
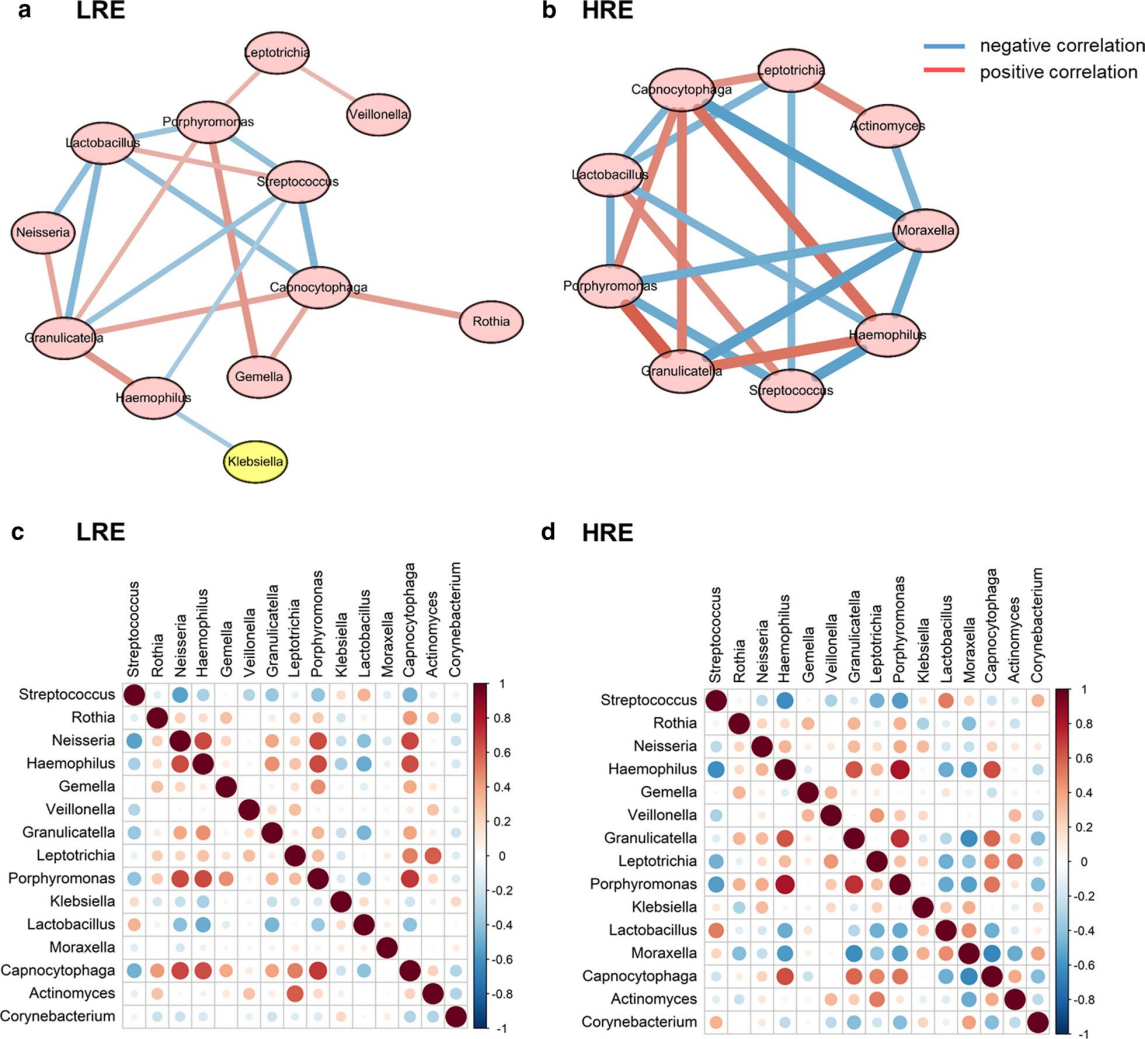
Sputum microbiome networks in COPD patients in different phenotypic subgroups. The networks of the top 15 genera were built using Spearman correlation coefficients in COPD patients. In total, 12 and 9 nodes were constructed in the LRE (**a**) and HRE groups (**b**), respectively. The nodes represent bacterial genera. The red and blue lines represent positive and negative correlations, respectively. Heatmap of the Spearman correlation matrix from the LRE (**c**) or HRE groups (**d**) with the top 15 genera. In the figure, the larger the circle, the higher is the correlation coefficient. Red dots represent positive correlations, and blue dots represent negative correlations. The correlation values ranged from − 1.00 (blue) to 1.00 (red). *LRE* low-risk exacerbator, *HRE* high-risk exacerbator

To explore the potential bacterial co-existence and co-exclusion relationships, we performed an interaction network analysis. We first selected the top 15 most abundant genera in each group, and the specific network was built and estimated based on the relative abundances of bacterial genera using SparCC correlation coefficients. Each node represents a genus of bacteria, and the red and blue lines represent positive and negative correlations, respectively. All plotted nodes of the networks with significant coefficients are shown in Fig. 4. In total, 12 and 9 nodes were constructed in the LRE (Fig. 4a) and HRE groups (Fig. 4b), respectively. In HRE subjects, *Moraxella* was included in a closed negatively correlated network containing *Streptococcus*, *Haemophilus*, *Moraxella*, *Capnocytophaga*, *Lactobacillus*, and *Porphyromonas.* (Fig. 4b). Furthermore, *Actinomyces* showed a negative correlation with *Moraxella* in HRE subjects (Fig. 4b). The heat map of Spearman correlation coefficients between the top 15 genera is shown in the LRE and HRE groups (Fig. 4c, d).

**Fig. 5:**
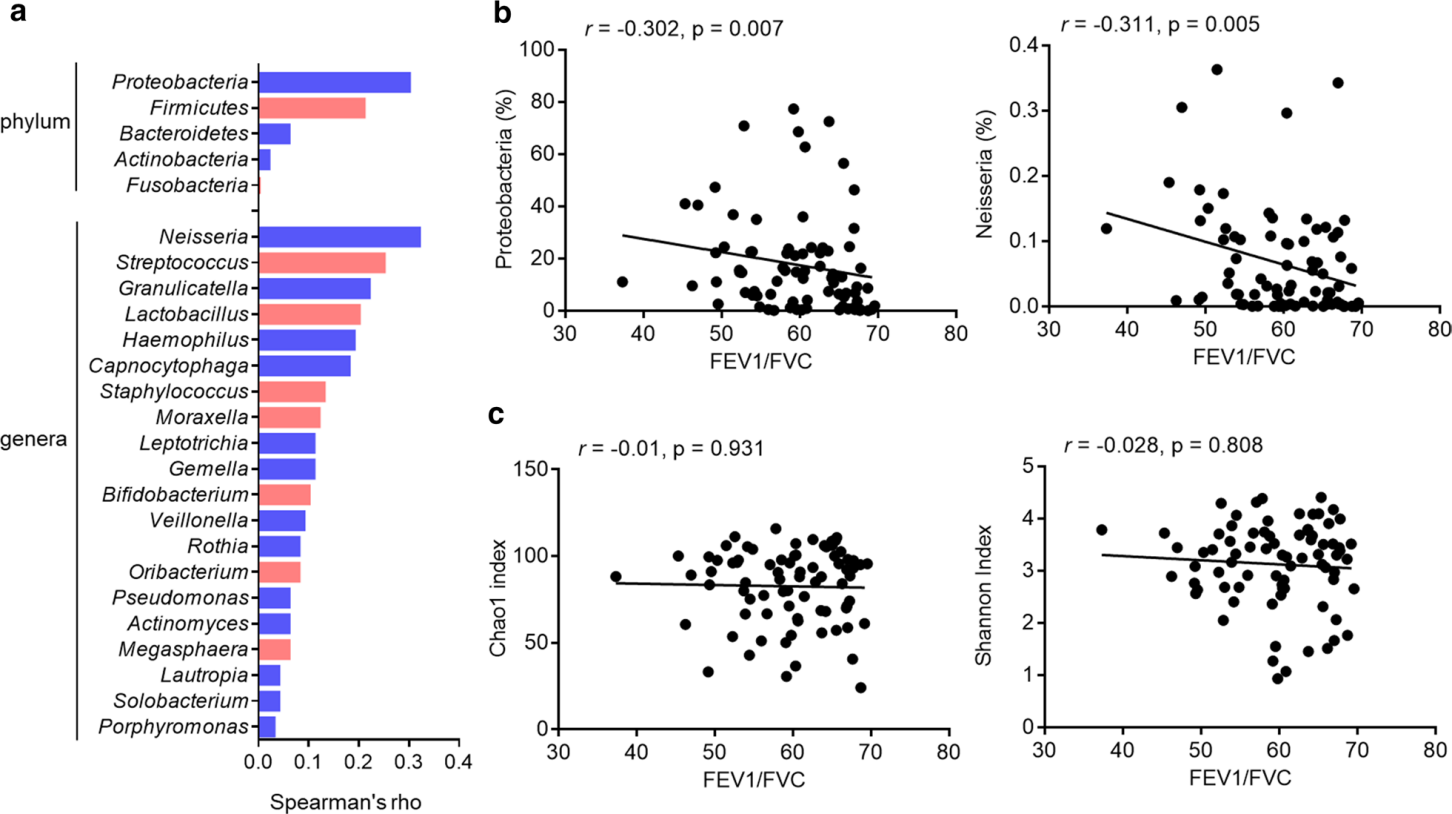
Correlation between the sputum microbiome and lung function in stable COPD. **a** Spearman correlations between the relative abundances of bacteria and the values of post-BD FEV1/FVC were calculated and plotted. **b** *Proteobacteria* and *Neisseria* show negative correlations with the values of post-BD FEV1/FVC. **c** The association of the Chao1 and Shannon indices and the values of post-BD FEV1/FVC. Bonferroni-adjusted p-values < 0.05/27 (5 phyla, 20 genera, 2 diversity indices) = 0.0019 indicate significance

To explore the potential relationship between different bacterial taxa and lung function in COPD patients, we performed Spearman’s correlation analyses using the 16S rRNA gene sequence dataset. Among the most abundant phyla and genera, the relative abundances of *Proteobacteria* and *Neisseria* were negatively correlated with FEV1/FVC (Fig. 5a, b). In a comparison of the Chao1 and Shannon indices with the FEV1/FVC value, there was no significant association with bacterial diversity and lung function (Fig. 5c).

### Functional analysis of the microbiome in stable COPD patients by PICRUSt analysis

**Fig. 6:**
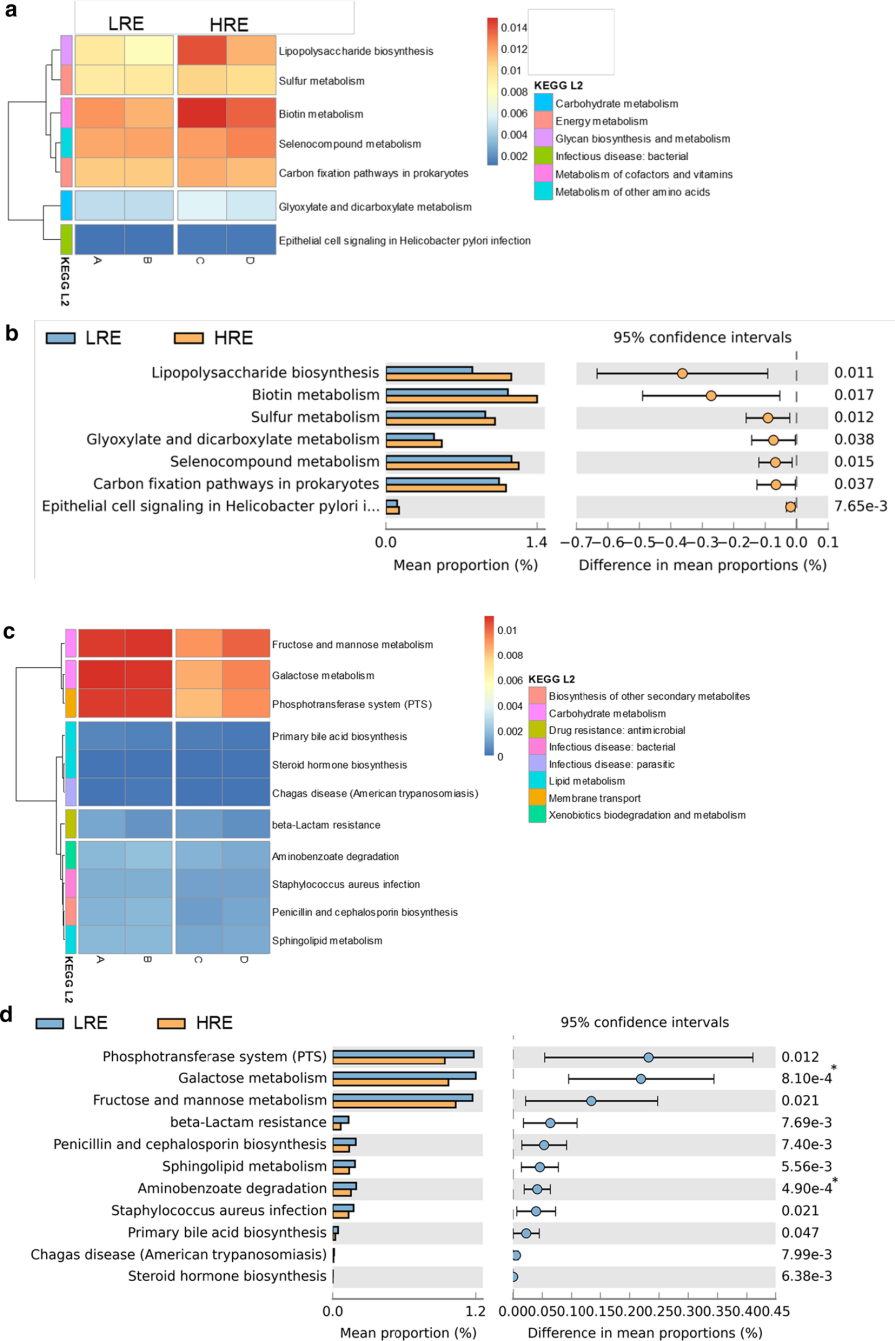
Differentially enriched functions in COPD patients by PICRUSt analysis. Comparison of the relative abundance of the PICRUSt-generated functional profile of the sputum microbiome in COPD patients. Upregulated KEGG pathways associated with HRE subjects are plotted as a heatmap (**a**) and bar graph (**b**). Bonferroni-adjusted p-values < 0.05/7 = 0.0071 indicate significance. Upregulated KEGG pathways associated with LRE subjects are plotted as a heatmap (**c**) and bar graph (**d**). Bonferroni-adjusted p-values < 0.05/11 = 0.0045 indicate significance. GOLD A, B, C, and D were identified via the GOLD 2017 classification. *LRE* low-risk exacerbator, *HRE* high-risk exacerbator

To explore the predicted functional capacity of the microbiome involved in COPD, we performed a PICRUSt analysis to predict the sputum microbiome functions in stable COPD patients. The results predicted a number of KEGG pathways that were slightly enriched or depleted associated with COPD exacerbation risk. The levels of metabolism, such as glycan biosynthesis and metabolism, lipopolysaccharide biosynthesis, sulphur metabolism, and biotin metabolism, were positively associated with high exacerbation risk of COPD, as their abundances were higher in the HRE group than in the LRE group (Fig. 6a, b). In contrast, parameters related to the phosphotransferase system, fructose and mannose metabolism and galactose metabolism were increased in the LRE group (Fig. 6c, d). These results may suggest that an altered sputum microbiome affect the nutrient availability, sugar metabolism, or growth conditions.

## Discussion

In 2017, the GOLD announced another major revision of the COPD guidelines. The recommendations for medications used in each patient group were also updated [8]. In this study, we extensively investigated the sputum microbiota in stable COPD patients with a high-throughput 16S rRNA gene sequencing analysis. Overall, the microbiome diversity decreased in HRE subjects, and the composition of the sputum microbiome changed between the two different exacerbation risk subgroups. We further explored the significant taxa and the predicted functional analyses associated with different COPD phenotypic subgroups. Collectively, our findings suggest notable airway microbiome changes in stable COPD patients and highlight its cooperative network and functional capacity.

Our results demonstrated that the bacterial richness was reduced in HRE COPD patients with even during periods of clinical stability. Mayhew et al*.* reported that the bacterial diversity index was reduced in very severe COPD patients compared with moderate COPD patients in sputum samples obtained in both stable and exacerbated states [16]. Diao et al*.* also reported that the OTU richness in throat swab samples from COPD patients was lower than that observed in samples obtained from healthy controls [29]. These results are consistent with our findings in this study. In contrast, Pragman et al*.* reported an increase in the microbial diversity index with the development of COPD in bronchoalveolar lavage fluid (BALF) samples in a cohort that included 14 moderate and 8 severe COPD patients [30]. These discrepancies may be due to the different geographical areas and the different sampling methods used.

In our study, the most dominant phylum in sputum samples was *Firmicutes* (approximately 43.5–54.6%), which was consistent with other reports that analysed sputum samples and identified the proportion of *Firmicutes* as approximately 40–50% [16, 31]. Our finding contrasts with the results of Garcia-Nunez et al*.* who also analysed sputum samples during stable COPD and observed that *Proteobacteria* (44%) and *Firmicutes* (16%) were the first and second most abundant phyla, respectively, in 17 moderate to advanced COPD patients [32]. The most dominant phylum in bronchial wash samples from stable COPD patients was *Firmicutes* [33]. Otherwise, *Bacteroides* (approximately 40–60%) was the most abundant phylum in lung and throat swab samples [29, 34]. There are differences in the microbiome at different locations within the respiratory tract [35]. The microbiota of the induced sputum might represent the upper airway microbiota, which would explain the difference in the microbiome compositions from the BALF or lung samples. Pragman et al*.* profiled the microbiome in oral, bronchial, and lung tissue samples from individual patients and observed that oral bacteria are true members of the early-stage COPD lung microbiota and exhibit ecological drift [36].

In the present study, the top 7 dominant genera present in COPD patient sputum samples were *Streptococcus*, *Rothia*, *Haemophilus*, *Neisseria*, *Veillonella*, *Granulicatella*, and *Porphyromonas*, genera to which some pathogenic bacteria belong*. Streptococcus* was the most common genus in the oral, bronchial, and lung tissue samples in COPD patients [36]. Of the 5 dominant genera identified from sputum samples by Mayhew et al. our study identified 3 common taxa (*Streptococcus*, *Haemophilus*, and *Veillonella*) among our 7 most common taxa. In the report by Tangedal et al*.* the 7 most dominant genera in induced sputum samples were identical to those observed in our results [37]. Furthermore, the relative abundance of *Streptococcus* was decreased in the stable HRE COPD patients compared with that in the LRE patients in our cohort. In contrast, the relative abundance of *Streptococcus* was not altered between the groups with different lung function levels. *Streptococcus pneumoniae* infection is reported to be important pathological bacteria in COPD in association with disease exacerbation or airway limitation [38]. However, the 16S rRNA sequencing platform was unable to discriminate between the various species in the genus of *Streptococcus* because the sequences of 16S rRNA gene in this genus are relatively similar.

Some reports have indicated that bacterial diversity was positively associated with lung function [30, 32], whereas other researchers observed that the bacterial diversity was not correlated with lung function [29]. In our study, the Chao1 diversity index and the observed OTUs were not associated with lung function (FEV1/FVC) in the 78 stable COPD patients. Another interesting finding from our study was that the proportions of *Proteobacteria* and *Neisseria* were negatively correlated with FEV1/FVC in stable COPD patients. The proportion of *Neisseria* was slightly increased in the HRE group (3.42%) compared to that in the LRE group (3.07%) in our cohort. *Neisseria* are gram-negative bacteria that belong to the family *Neisseriaceae* and are present in mucosal surfaces in the upper respiratory and genitourinary tracts [39]. Commensal *Neisseria* can be opportunistic pathogens in humans, and some clinical cases of infections with *Neisseria* species such as *N. bacilliformis* in sputum samples from patients with bronchitis and with *N. flavescens*, *N. lactamase,* and *N. mucosa* in lung samples from patients with pneumonia have been reported [40, 41].

The proportions of *Gemella morbillorum* (*G. morbillorum*, LDA score in LRE = 3.64, p value = 0.033) and *Prevotella histicola* (*P. histicola*, LDA score in LRE = 3.54, p value = 0.016) were significantly decreased in HRE subjects in the present study. *G. morbillorum* and *P. histicola* are some of the normal flora of the mucous membranes, predominantly of the oropharynx, but can also be found in the upper respiratory and other sites. *P. histicola* may suppress the production of inflammatory cytokines, and *P. histicola* suppresses disease in the animal model of multiple sclerosis or arthritis [42, 43]. These results may suggest that the altered normal flora distribution may affect the colonization of pathogenic bacteria and enhanced inflammation, leading to airway obstruction and increased exacerbation risk.

Functional prediction showed that lipopolysaccharides (LPS) biosynthesis, which produces the main cell wall components of gram-negative bacteria, was slightly enriched in HRE subjects. LPS challenging may lead to air flow limitation, decreases in the level of FEV1 and enhanced pulmonary inflammation, suggesting that this occurs during exacerbations in COPD patients [44, 45]. Also, sulphur and biotin metabolism were enriched in HRE subjects [46, 47]. These pathways are essential for the survival of bacteria, including some pathogens. Furthermore, the levels of phosphotransferase system-related parameters, such as fructose and mannose metabolism and galactose metabolism were significantly reduced in COPD patients compared to the controls. The phosphotransferase system plays a pivotal role in the uptake of multiple sugars in bacteria. Glucose concentration in the airway might contribute to bacterial infections, and impaired glucose metabolism was also observed in COPD [48, 49]. These results may suggest that the normal flora balance is disrupted and that the energy and metabolic machinery of normal and/or pathogenic bacteria are altered.

An important limitation of our study was that no follow-up was conducted. Further studies with longitudinal sampling from each individual at both stable and exacerbation time points will be important for monitoring the microbiome dynamics, clinical phenotypes and treatment responses. Another limitation of our study was that the patients were enrolled at a single site; therefore, exploring other variables between different areas was not possible. A third limitation was that fewer female subjects were enrolled. We observed that among the PFT II group (n = 35), only about 40% of patients (n = 14) had severe airflow limitation (FEV1 < 50) and frequent exacerbations, although this finding could be due to the small sample size of this subgroup in our cohort during the one-year study. Interestingly, it was previously reported that “high-risk” COPD patients (GOLD groups C and D) are highly heterogeneous populations [50]. Moreover, environmental and occupational exposures may affect the exacerbation of COPD patients.

## Conclusions

In conclusion, the present study revealed that the sputum microbiome changed in different exacerbation risk subgroups of COPD. Additionally, the bacterial cooperative networks were different in the different COPD phenotypic subgroups. An altered lung microbiome can have an important effect on the host immunity and initiates disease pathogenesis, promotes chronic inflammation, or merely serves as a marker of injury and inflammation. Understanding the mechanisms driving bacterial compositions and diseases will help us prevent or treat COPD.

## Supplementary Information

**Additional file 1.** 16S rRNA gene sequencing of sputum microbiome in COPD.

## Data Availability

The datasets were deposited into the NCBI Sequence Read Archive (SRA) database with the project number PRJNA636302.

## References

[1] Collaborators GBDCRD (2017). Global, regional, and national deaths, prevalence, disability-adjusted life years, and years lived with disability for chronic obstructive pulmonary disease and asthma, 1990–2015: a systematic analysis for the Global Burden of Disease Study 2015. Lancet Respir Med.

[2] Lim S, Lam DC, Muttalif AR, Yunus F, Wongtim S, le Lan TT, Shetty V, Chu R, Zheng J, Perng DW (2015). Impact of chronic obstructive pulmonary disease (COPD) in the Asia-Pacific region: the EPIC Asia population-based survey. Asia Pac Fam Med.

[3] Papaioannou AI, Loukides S, Gourgoulianis KI, Kostikas K (2009). Global assessment of the COPD patient: time to look beyond FEV1?. Respir Med.

[4] Soriano JB, Lamprecht B, Ramirez AS, Martinez-Camblor P, Kaiser B, Alfageme I, Almagro P, Casanova C, Esteban C, Soler-Cataluna JJ (2015). Mortality prediction in chronic obstructive pulmonary disease comparing the GOLD 2007 and 2011 staging systems: a pooled analysis of individual patient data. Lancet Respir Med.

[5] McGhan R, Radcliff T, Fish R, Sutherland ER, Welsh C, Make B (2007). Predictors of rehospitalization and death after a severe exacerbation of COPD. Chest.

[6] Hurst JR, Vestbo J, Anzueto A, Locantore N, Mullerova H, Tal-Singer R, Miller B, Lomas DA, Agusti A, Macnee W (2010). Susceptibility to exacerbation in chronic obstructive pulmonary disease. N Engl J Med.

[7] Vestbo J, Hurd SS, Agusti AG, Jones PW, Vogelmeier C, Anzueto A, Barnes PJ, Fabbri LM, Martinez FJ, Nishimura M (2013). Global strategy for the diagnosis, management, and prevention of chronic obstructive pulmonary disease: GOLD executive summary. Am J Respir Crit Care Med.

[8] Vogelmeier CF, Criner GJ, Martinez FJ, Anzueto A, Barnes PJ, Bourbeau J, Celli BR, Chen R, Decramer M, Fabbri LM (2017). Global strategy for the diagnosis, management, and prevention of chronic obstructive lung disease 2017 report. GOLD executive summary. Am J Respir Crit Care Med.

[9] Agusti A, Vestbo J (2011). Current controversies and future perspectives in chronic obstructive pulmonary disease. Am J Respir Crit Care Med.

[10] Garcha DS, Thurston SJ, Patel AR, Mackay AJ, Goldring JJ, Donaldson GC, McHugh TD, Wedzicha JA (2012). Changes in prevalence and load of airway bacteria using quantitative PCR in stable and exacerbated COPD. Thorax.

[11] Han MK, Huang YJ, Lipuma JJ, Boushey HA, Boucher RC, Cookson WO, Curtis JL, Erb-Downward J, Lynch SV, Sethi S (2012). Significance of the microbiome in obstructive lung disease. Thorax.

[12] Huang YJ, Sethi S, Murphy T, Nariya S, Boushey HA, Lynch SV (2014). Airway microbiome dynamics in exacerbations of chronic obstructive pulmonary disease. J Clin Microbiol.

[13] Wilkinson TM, Patel IS, Wilks M, Donaldson GC, Wedzicha JA (2003). Airway bacterial load and FEV1 decline in patients with chronic obstructive pulmonary disease. Am J Respir Crit Care Med.

[14] Sethi S, Evans N, Grant BJ, Murphy TF (2002). New strains of bacteria and exacerbations of chronic obstructive pulmonary disease. N Engl J Med.

[15] Segal LN, Rom WN, Weiden MD (2014). Lung microbiome for clinicians. New discoveries about bugs in healthy and diseased lungs. Ann Am Thorac Soc.

[16] Mayhew D, Devos N, Lambert C, Brown JR, Clarke SC, Kim VL, Magid-Slav M, Miller BE, Ostridge KK, Patel R (2018). Longitudinal profiling of the lung microbiome in the AERIS study demonstrates repeatability of bacterial and eosinophilic COPD exacerbations. Thorax.

[17] Wang Z, Singh R, Miller BE, Tal-Singer R, Van Horn S, Tomsho L, Mackay A, Allinson JP, Webb AJ, Brookes AJ (2018). Sputum microbiome temporal variability and dysbiosis in chronic obstructive pulmonary disease exacerbations: an analysis of the COPDMAP study. Thorax.

[18] Pragman AA, Knutson KA, Gould TJ, Isaacson RE, Reilly CS, Wendt CH (2019). Chronic obstructive pulmonary disease upper airway microbiota alpha diversity is associated with exacerbation phenotype: a case-control observational study. Respir Res.

[19] Pavord ID, Pizzichini MM, Pizzichini E, Hargreave FE (1997). The use of induced sputum to investigate airway inflammation. Thorax.

[20] Yang CY, Yeh YM, Yu HY, Chin CY, Hsu CW, Liu H, Huang PJ, Hu SN, Liao CT, Chang KP (2018). Oral microbiota community dynamics associated with oral squamous cell carcinoma staging. Front Microbiol.

[21] Magoc T, Salzberg SL (2011). FLASH: fast length adjustment of short reads to improve genome assemblies. Bioinformatics.

[22] Caporaso JG, Kuczynski J, Stombaugh J, Bittinger K, Bushman FD, Costello EK, Fierer N, Pena AG, Goodrich JK, Gordon JI (2010). QIIME allows analysis of high-throughput community sequencing data. Nat Methods.

[23] Edgar RC, Haas BJ, Clemente JC, Quince C, Knight R (2011). UCHIME improves sensitivity and speed of chimera detection. Bioinformatics.

[24] Wang Q, Garrity GM, Tiedje JM, Cole JR (2007). Naive Bayesian classifier for rapid assignment of rRNA sequences into the new bacterial taxonomy. Appl Environ Microbiol.

[25] Callahan BJ, McMurdie PJ, Rosen MJ, Han AW, Johnson AJ, Holmes SP (2016). DADA2: High-resolution sample inference from Illumina amplicon data. Nat Methods.

[26] Sam Ma Z, Guan Q, Ye C, Zhang C, Foster JA, Forney LJ (2015). Network analysis suggests a potentially 'evil' alliance of opportunistic pathogens inhibited by a cooperative network in human milk bacterial communities. Sci Rep.

[27] Segata N, Izard J, Waldron L, Gevers D, Miropolsky L, Garrett WS, Huttenhower C (2011). Metagenomic biomarker discovery and explanation. Genome Biol.

[28] Parks DH, Tyson GW, Hugenholtz P, Beiko RG (2014). STAMP: statistical analysis of taxonomic and functional profiles. Bioinformatics.

[29] Diao W, Shen N, Du Y, Qian K, He B (2017). Characterization of throat microbial flora in smokers with or without COPD. Int J Chron Obstruct Pulmon Dis.

[30] Pragman AA, Kim HB, Reilly CS, Wendt C, Isaacson RE (2012). The lung microbiome in moderate and severe chronic obstructive pulmonary disease. PLoS ONE.

[31] Wang Z, Bafadhel M, Haldar K, Spivak A, Mayhew D, Miller BE, Tal-Singer R, Johnston SL, Ramsheh MY, Barer MR (2016). Lung microbiome dynamics in COPD exacerbations. Eur Respir J.

[32] Garcia-Nunez M, Millares L, Pomares X, Ferrari R, Perez-Brocal V, Gallego M, Espasa M, Moya A, Monso E (2014). Severity-related changes of bronchial microbiome in chronic obstructive pulmonary disease. J Clin Microbiol.

[33] Einarsson GG, Comer DM, McIlreavey L, Parkhill J, Ennis M, Tunney MM, Elborn JS (2016). Community dynamics and the lower airway microbiota in stable chronic obstructive pulmonary disease, smokers and healthy non-smokers. Thorax.

[34] Sze MA, Dimitriu PA, Suzuki M, McDonough JE, Campbell JD, Brothers JF, Erb-Downward JR, Huffnagle GB, Hayashi S, Elliott WM (2015). Host response to the lung microbiome in chronic obstructive pulmonary disease. Am J Respir Crit Care Med.

[35] Dickson RP, Erb-Downward JR, Martinez FJ, Huffnagle GB (2016). The microbiome and the respiratory tract. Annu Rev Physiol.

[36] Pragman AA, Lyu T, Baller JA, Gould TJ, Kelly RF, Reilly CS, Isaacson RE, Wendt CH (2018). The lung tissue microbiota of mild and moderate chronic obstructive pulmonary disease. Microbiome.

[37] Tangedal S, Aanerud M, Gronseth R, Drengenes C, Wiker HG, Bakke PS, Eagan TM (2017). Comparing microbiota profiles in induced and spontaneous sputum samples in COPD patients. Respir Res.

[38] Beasley V, Joshi PV, Singanayagam A, Molyneaux PL, Johnston SL, Mallia P (2012). Lung microbiology and exacerbations in COPD. Int J Chron Obstruct Pulmon Dis.

[39] Liu G, Tang CM, Exley RM (2015). Non-pathogenic Neisseria: members of an abundant, multi-habitat, diverse genus. Microbiology.

[40] Zhou Y, Lin P, Li Q, Han L, Zheng H, Wei Y, Cui Z, Ni Y, Guo X (2010). Analysis of the microbiota of sputum samples from patients with lower respiratory tract infections. Acta Biochim Biophys Sin (Shanghai).

[41] Humbert MV, Christodoulides M (2019). Atypical, yet not infrequent, infections with *Neisseria* species. Pathogens.

[42] Shahi SK, Freedman SN, Murra AC, Zarei K, Sompallae R, Gibson-Corley KN, Karandikar NJ, Murray JA, Mangalam AK (2019). Prevotella histicola, a human gut commensal, is as potent as COPAXONE(R) in an animal model of multiple sclerosis. Front Immunol.

[43] Marietta EV, Murray JA, Luckey DH, Jeraldo PR, Lamba A, Patel R, Luthra HS, Mangalam A, Taneja V (2016). Suppression of inflammatory arthritis by human gut-derived prevotella histicola in humanized mice. Arthrit Rheumatol.

[44] Aul R, Armstrong J, Duvoix A, Lomas D, Hayes B, Miller BE, Jagger C, Singh D (2012). Inhaled LPS challenges in smokers: a study of pulmonary and systemic effects. Br J Clin Pharmacol.

[45] Moller W, Heimbeck I, Hofer TP, Khadem Saba G, Neiswirth M, Frankenberger M, Ziegler-Heitbrock L (2012). Differential inflammatory response to inhaled lipopolysaccharide targeted either to the airways or the alveoli in man. PLoS ONE.

[46] Kertesz MA (2000). Riding the sulfur cycle–metabolism of sulfonates and sulfate esters in gram-negative bacteria. FEMS Microbiol Rev.

[47] Rodionov DA, Mironov AA, Gelfand MS (2002). Conservation of the biotin regulon and the BirA regulatory signal in Eubacteria and Archaea. Genome Res.

[48] Mallia P, Webber J, Gill SK, Trujillo-Torralbo MB, Calderazzo MA, Finney L, Bakhsoliani E, Farne H, Singanayagam A, Footitt J (2018). Role of airway glucose in bacterial infections in patients with chronic obstructive pulmonary disease. J Allergy Clin Immunol.

[49] Agarwal AR, Kadam S, Brahme A, Agrawal M, Apte K, Narke G, Kekan K, Madas S, Salvi S (2019). Systemic Immuno-metabolic alterations in chronic obstructive pulmonary disease (COPD). Respir Res.

[50] Agusti A, Rennard S, Edwards LD, MacNee W, Wouters E, Miller B, Tal-Singer R, Mullerova H, Celli B (2015). Evaluation of CLtIPSEi: clinical and prognostic heterogeneity of C and D GOLD groups. Eur Respir J.

