# Supplementary material for: Association of exacerbation phenotype with the sputum microbiome in chronic obstructive pulmonary disease patients during the clinically stable state
Source: J Transl Med. 2021 Mar 23;19:121. doi: 10.1186/s12967-021-02788-4 (PMC7988976; doi:10.1186/s12967-021-02788-4)
Supplement: Supplementary file 1 — Additional file 1. 16S rRNA gene sequencing of sputum microbiome in COPD. [file 12967_2021_2788_MOESM1_ESM.docx]

**Additional information**

**Methods**

**Study subjects**

**Table S1. The inclusion and exclusion criteria.**

| **Low-risk Exacerbators (LRE)** | **Inclusion Criteria** | **Exclusion Criteria** |
| --- | --- | --- |
|  | 1. Age ≥ 40 years 2. post-bronchodilator (post-BD) FEV_1_/FVC ≤ 0.7 3. < 2 moderate exacerbations and no severe exacerbations per year | 1. Patients aged < 40 years 2. Asthma diagnosis 3. Cystic fibrosis 4. Neoplasia 5. Patients who underwent chronic treatment with oral corticosteroids or immunosuppressive drugs 6. Patients who were treated with systemic steroids or antibiotics in the previous 4 weeks |
| **High-risk Exacerbators (HRE)** | **Inclusion Criteria** | **Exclusion Criteria** |
|  | 1. Age ≥ 40 years 2. post-bronchodilator (post-BD) FEV_1_/FVC ≤ 0.7 3. ≥ 2 moderate or severe exacerbations or ≥ 1 hospitalizations for COPD exacerbation | 1. Patients aged < 40 years 2. Asthma diagnosis 3. Cystic fibrosis 4. Neoplasia 5. Patients who underwent chronic treatment with oral corticosteroids or immunosuppressive drugs 6. Patients who were treated with systemic steroids or antibiotics in the previous 4 weeks |

We categorized each patient in our cohort according to GOLD 2017 classifications. Patients had no exacerbations or infections for at least 30 days prior to sample collection, and their sociodemographic and clinical data were recorded (current medication, smoking history, number of exacerbations treated with systemic steroids or antibiotics and exacerbation-related hospital admissions in the preceding year). Overall the exclusion criteria were patients aged < 40 years; patients with a diagnosis of asthma, cystic fibrosis, or neoplasia; patients who underwent chronic treatment with oral corticosteroids or immunosuppressive drugs; and patients who were admitted to a hospital for respiratory symptoms in the previous 4 weeks. The enrolled COPD patients were further grouped as HRE or LRE based on the criteria described above.

**Sputum sampling and processing**

Spirometry was performed, and reversibility to salbutamol was tested in the COPD patients. Fifteen minutes after the inhalation of 200 μg of salbutamol, the patients rinsed their mouths with water. Spirometry was performed to assess reversibility to salbutamol, and oxygen saturation and pulse rate were measured using a pulse oximeter. Sputum induction consisted of inhaling an aerosol of saline produced from an ultrasonic nebulizer (ULTRAMIST KUN868). After 5 min of nebulization with 3% saline solution, the subjects were asked to expectorate into a sterile sputum pot. Lung function and oxygen saturation were recorded, with nebulization recommenced if there was a < 20% decrease in FEV1% predicated. If there was a > 20% decrease in FEV1% predicated, the procedure was stopped, and inhaled salbutamol was administered if FEV1% predicated was slow to return to baseline. This procedure was repeated every 5 min for a total nebulization period of 20 min. Sputum samples with a percentage of squamous cells higher than 20% were excluded. The sputum samples were stored at -80℃ until further analysis. Sputum induction was successful in about 87% of patients, and finally, a total of 78 patients was enrolled.

**Sputum microbiota profiling by 16S rRNA gene sequencing**

A 16S rRNA gene amplicon library targeting the 16S rRNA V3-V4 region was constructed as our previous report [13]. Illumina adaptor overhang nucleotide sequences were added to the gene-specific sequences (16S rRNA gene amplicon PCR forward primer sequence = 5’-TCGTCGGCAGCGTCAGATGTGTATAAGAGACAGCCTACGGGNGGCWG

CAG-3’ and 16S rRNA gene amplicon PCR reverse primer sequence = 5’- GTCTCGTGGGCTCGGAGATGTGTATAAGAGACAGGACTACHVGGGTATCTAATCC-3’). The first PCR mixture contained 1 μM of both forward and reverse primers, 1× KAPA HiFi Hotstart Ready Mix, and bacterial genomic DNA (10 ng). The first PCR condition consisted of 3 min of denaturation at 95°C followed by 25 cycles of denaturation at 95°C (30 seconds), annealing at 55°C (30 seconds), and extension at 72°C (30 seconds), with a final 72°C extension for 30 seconds. The PCR products were purified using AMPure XP beads, followed by index PCR. Each index PCR mixture contained 5 μl of both Nextera XT index primers 1 and 2, 1× KAPA HiFi Hotstart Ready Mix, and the purified products of the first PCR. The index PCR condition consisted of 3 min of denaturation at 95°C followed by 8 cycles of denaturation at 95°C (30 seconds), annealing at 55°C (30 seconds), and extension at 72°C (30 seconds), with a final extension at 72°C for 5 min. The final amplicon libraries were approximately 630 bp in length and were validated using the HT DNA High Sensitivity LabChip Kit (Caliper, Perkin-Elmer, MA, USA). The multiplex amplified libraries were pooled equally and sequenced on a MiSeq system with 2 × 300 paired-end v3 sequencing reagents (Illumina, USA).

**Bioinformatics analysis**

The sequencing reads were initially demultiplexed using MiSeq Reporter v2.6 according to the sample barcodes. The resulting pairs of reads from each sample were merged to obtain longer reads (460 ± 50 bp) to improve the taxonomic classification using FLASH (V1.2.11)^1^. Low-quality reads with q-values < 20 were filtered by the split_libraries_fastq.py script in QIIME (Version 1.9.1)^2^. The package Cutadapt v1.14 was used to remove forward and reverse sequencing primers from the merged reads of each dataset. The resulting sequence tags were compared to the GOLD database (http://drive5.com/uchime/gold.fa) to remove chimaeric sequences using UCHIME. Only sequence tags with lengths of > 400 bp were retained for subsequent analysis. The operational taxonomic units (OTUs) were clustered at 97% sequence similarity using USEARCH (v9.2.64)^3^ against the Greengenes 16S rRNA gene database (13_8 release), and final taxonomic assignments were performed using RDP-classifier^4^. Furthermore, our results were validated by another bioinformatics pipeline using the DADA2 package^5^ for modelling and amplicon error correction, which was followed by quality filtering, dereplication, denoising, merging and chimaera removal. Trimming and filtering were performed on paired reads jointly, and both reads were required to pass the filter for the pair to pass. Therefore, the filtered read numbers were the same for R1 and R2. Forward and reverse sequences at positions 290 and 220 were truncated, and the first 13 bases of each sequences were trimmed. All other DADA2 parameters retained their default settings. A Naïve Bayes classifier^4^ was trained using the most recent available version of the Silva (version 132) sequences for taxonomic assignments. A multiple-alignment was performed using the DECIPHER R package, and the phangorn R package was then used to construct a phylogenetic tree. A prevalence filter used to remove rare taxa was set at 5% of total samples. According to the 16S rRNA gene bacterial profiling data, we performed a bivariate correlation analysis of the 15 most abundant genera using Spearman’s correlation coefficient in R. We then constructed a co-occurrence network of the predominant sputum microbiota with different COPD disease severities. The network was generated using Cytoscape (version 3.7.0) and visualized using a circular layout in which the nodes represented bacterial genera and the edges represented the strength of the positive (red) or negative (blue) correlation between the genera with the widths of the edges reflected by the Spearman’s correlation values^6^. Potential biomarkers were determined using the linear discriminate analysis effect size (LEfSe)^7^, which revealed the significant differences of sputum microbiota compositions among COPD disease severities. LEfSE was performed to illustrate differences using the Kruskal-Wallis (KW) sum-rank test and pairwise Wilcoxon rank-sum test, with linear discriminant analysis (LDA) of greater than 2.0. The microbial functionality profiles were predicted using a Phylogenetic Investigation of Communities by Reconstruction of Unobserved States (PICRUSt) to generate the Kyoto Encyclopedia of Genes and Genomes (KEGG) pathways. The predicted genes and their functions were aligned to the KEGG database, and the differences among groups were compared using structural time series analyser, modeller, and predictor (STAMP) (version 2.1.3)^8^.

References

1. Magoc T, Salzberg SL: FLASH: fast length adjustment of short reads to improve genome assemblies. *Bioinformatics* 2011, 27(21):2957-2963.
2. Caporaso JG, Kuczynski J, Stombaugh J, Bittinger K, Bushman FD, Costello EK, Fierer N, Pena AG, Goodrich JK, Gordon JI *et al*: QIIME allows analysis of high-throughput community sequencing data. *Nat Methods* 2010, 7(5):335-336.
3. Edgar RC, Haas BJ, Clemente JC, Quince C, Knight R: UCHIME improves sensitivity and speed of chimera detection. *Bioinformatics* 2011, 27(16):2194-2200.
4. Wang Q, Garrity GM, Tiedje JM, Cole JR: Naive Bayesian classifier for rapid assignment of rRNA sequences into the new bacterial taxonomy. *Appl Environ Microbiol* 2007, 73(16):5261-5267.
5. Callahan BJ, McMurdie PJ, Rosen MJ, Han AW, Johnson AJ, Holmes SP: DADA2: High-resolution sample inference from Illumina amplicon data. *Nat Methods* 2016, 13(7):581-583.
6. Sam Ma Z, Guan Q, Ye C, Zhang C, Foster JA, Forney LJ: Network analysis suggests a potentially 'evil' alliance of opportunistic pathogens inhibited by a cooperative network in human milk bacterial communities. *Sci Rep* 2015, 5:8275.
7. Segata N, Izard J, Waldron L, Gevers D, Miropolsky L, Garrett WS, Huttenhower C: Metagenomic biomarker discovery and explanation. *Genome Biol* 2011, 12(6):R60.
8. Parks DH, Tyson GW, Hugenholtz P, Beiko RG: STAMP: statistical analysis of taxonomic and functional profiles. *Bioinformatics* 2014, 30(21):3123-3124.

**Table S2. The statistic results of 16S sequencing in 78 COPD patients.**

|  | All | LRE | HRE | PFT I | PFT II |
| --- | --- | --- | --- | --- | --- |
| Number of patients | 78 | 60 | 18 | 43 | 35 |
| Raw paired reads |  |  |  |  |  |
| range | 56,784 ~ 450,590 | 56,784 ~ 450,590 | 75,359 ~ 416,144 | 56,784 ~ 450,590 | 75,359 ~ 424,668 |
| mean ± SD | 228,741 ± 104,073 | 224,394 ± 105,973 | 243,230 ± 98,965 | 228,169 ± 107,429 | 229,443 ± 101,351 |
| Combined paired reads |  |  |  |  |  |
| range | 35,718 ~ 336,162 | 35,718 ~ 336,162 | 48,885 ~ 318,709 | 35,718 ~ 336,162 | 48,885 ~ 328,294 |
| mean ± SD | 162,469 ± 85,914 | 158,041 ± 86,979 | 177,230 ± 82,924 | 160,068 ± 86,772 | 165,419 ± 86,019 |
| Quality-filtered reads |  |  |  |  |  |
| range | 32,419 ~ 273,280 | 32,419 ~ 273,280 | 44,019 ~ 250,231 | 32,419 ~ 273,280 | 44,019 ~ 259,641 |
| mean ± SD | 133,386 ± 64,433 | 129,691 ± 65,147 | 145,703 ± 62,176 | 131,481 ± 65,045 | 135,726 ± 64,541 |

Data are presented in range or mean ± standard deviation.

LRE: Low risk exacerbator; HRE: High risk exacerbator; PFT: pulmonary function test; PFT I: FEV1 ≥ 50; PFT II: FEV1 < 50; FEV1, forced expiratory volume in the first second

**Table S3. Taxonomic identification at the phylum and genus levels in old-aged COPD.**

| Patients > 60 years | LRE (n = 51) | HRE (n = 16) | p value |
| --- | --- | --- | --- |
| **Phylum** |  |  |  |
| Firmicutes | 56.32 ± 2.77 | 45.49 ± 4.50 | 0.046 |
| Actinobacteria | 19.44 ±1.88 | 16.36 ± 1.97 | 0.607 |
| Proteobacteria | 15.46 ± 2.64 | 26.49 ± 5.15 | 0.023 |
| Bacteroidetes | 4.21 ± 0.62 | 5.65 ± 1.44 | 0.659 |
| Fusobacteria | 2.89 ± 0.43 | 5.02 ± 1.80 | 0.746 |
| **Genera** |  |  |  |
| Streptococcus | 44.57 ± 2.62 | 30.93 ± 3.8 | 0.004 |
| Rothia | 14.43 ± 1.58 | 12.61 ± 2.25 | 0.691 |
| Neisseria | 5.95± 1.03 | 8.27 ± 2.77 | 0.499 |
| Haemophilus | 4.22 ± 0.68 | 8.97 ± 4.10 | 0.941 |
| Granulicatella | 3.36 ± 0.52 | 3.52 ± 0.67 | 0.489 |
| Veillonella | 3.01 ± 0.56 | 5.76 ± 2.42 | 0.362 |
| Leptotrichia | 2.12 ± 0.38 | 4.80 ± 2.72 | 0.780 |
| Porphyromonas | 1.85 ± 0.41 | 2.77 ± 1.07 | 0.701 |
| Actinomyces | 0.94 ± 0.16 | 1.47 ± 0.45 | 0.343 |
| Capnocytophaga | 1.00 ± 0.25 | 2.06 ± 0.99 | 0.768 |

Data are presented as mean ± s.e.m. LRE: Low risk exacerbator; HRE: High risk exacerbator

The p value is analyzed using Mann Whitney Wilcoxon test. Bonferroni-adjusted p-values < 0.05/15 (5 phylum and 10 genus) = 0.0033 indicate significance.

**Table S4. Taxonomic identification at the phylum and genus levels in smoker vs non-smoker COPD.**

| Phylum | smoker | nonsmoker | p value |
| --- | --- | --- | --- |
| Patients | 61 | 17 |  |
| Firmicutes | 53.32 ± 2.59 | 56.97 ± 3.95 | 0.348 |
| Actinobacteria | 18.4 ± 1.6 | 17.4 ± 2.16 | 0.966 |
| Proteobacteria | 18.54 ± 2.46 | 14.3 ± 4.25 | 0.361 |
| Bacteroidetes | 4.94 ± 0.63 | 4.53 ± 1.1 | 0.995 |
| Fusobacteria | 3.2 ± 0.46 | 4.46 ± 1.41 | 0.457 |
| Genera | smoker | nonsmoker | p value |
| Streptococcus | 41.68 ± 2.52 | 40.53 ± 4.11 | 0.966 |
| Rothia | 13.36 ± 1.37 | 12.46 ± 2.13 | 0.937 |
| Neisseria | 7.2 ± 1.06 | 4.11 ± 1.69 | 0.243 |
| Haemophilus | 6.10 ± 1.24 | 3.86 ± 1.31 | 0.694 |
| Granulicatella | 3.14 ± 0.4 | 5.80 ± 0.75 | 0.193 |
| Veillonella | 2.74 ± 0.39 | 6.00 ± 2.46 | 0.624 |
| Leptotrichia | 2.29 ± 0.42 | 4.19 ± 2.41 | 0.387 |
| Porphyromonas | 2.28 ± 0.43 | 2.18 ± 0.74 | 0.73 |
| Actinomyces | 0.91 ± 0.13 | 1.72 ± 0.49 | 0.248 |
| Capnocytophaga | 1.37 ± 0.33 | 1.06 ± 0.38 | 0.899 |

The p value is analyzed using Mann Whitney Wilcoxon test. Bonferroni-adjusted p-values < 0.05/15 (5 phylum and 10 genus) = 0.0033 indicate significance.

**Additional Figures**

**
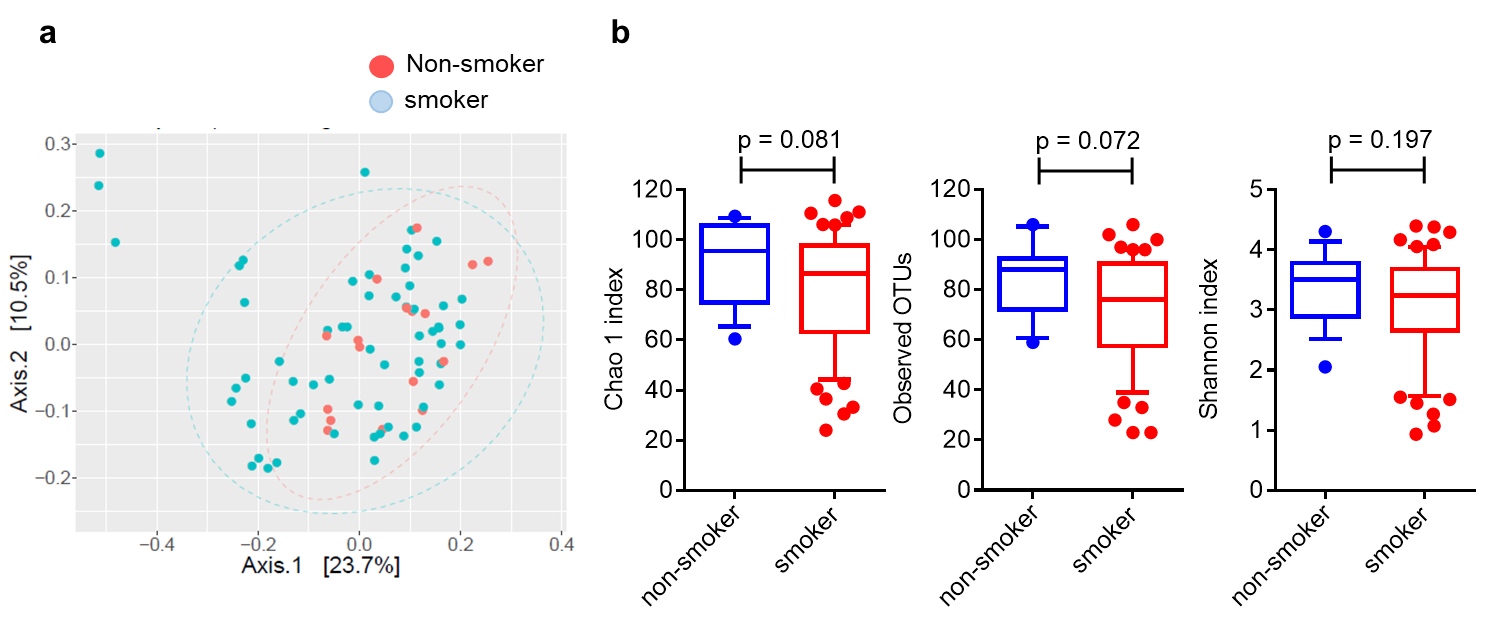
**

**Figure S1. The principal component analysis and diversity plot in case with non-smoker and smoker.**

The unweighted PCA plot (**a**) and Chao1 index, observed OTUs, Shannon index (**b**) are calculated in non-smoker vs smoker COPD patients. The box and whiskers plots shows the median, 10th and 90th percentile in each group. Bonferroni-adjusted p-values < 0.05/3 = 0.0167 indicate significance.

**
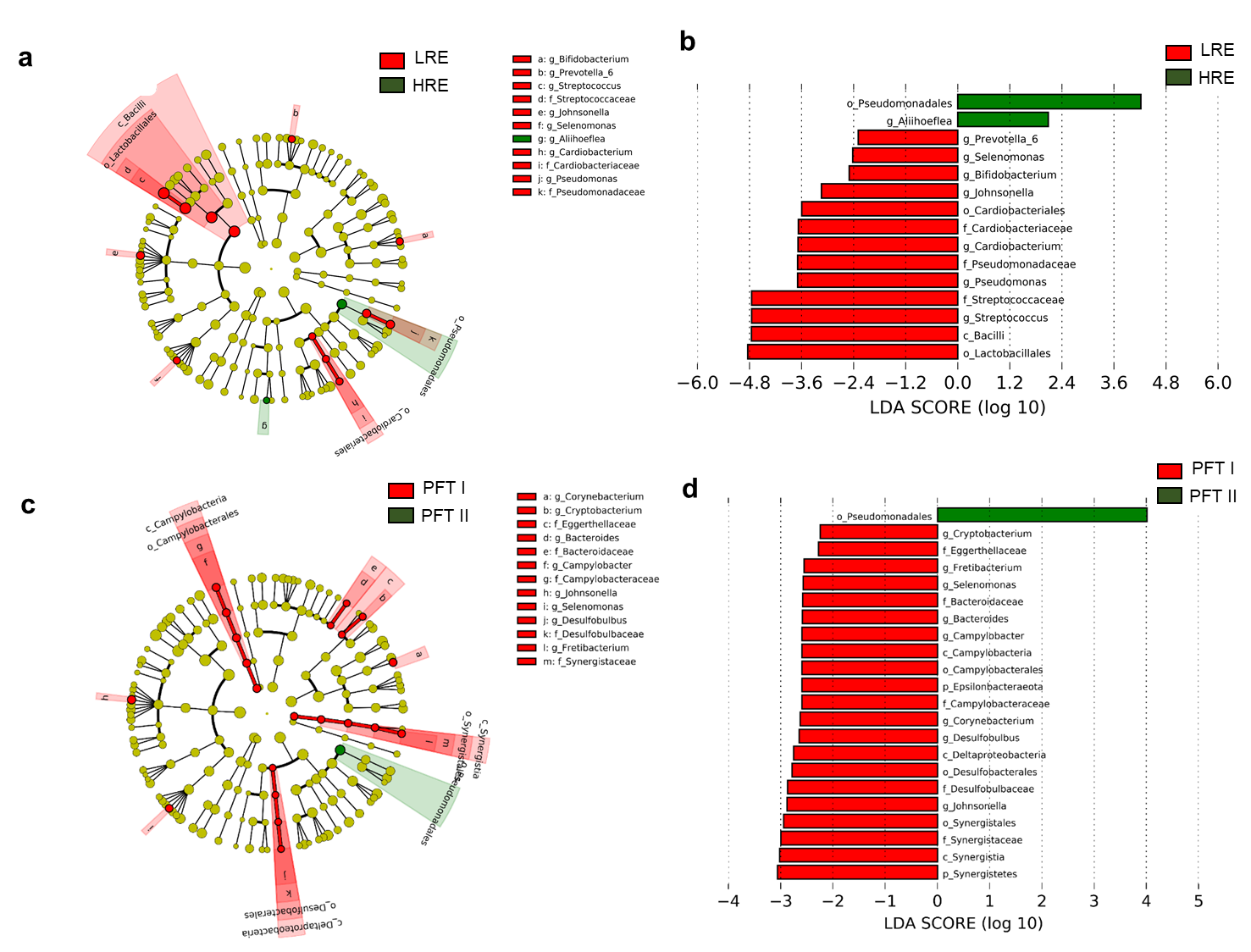
**

**Figure S2. Differentially enriched bacterial taxa in COPD patients by LEfSe analysis.** Circular taxonomic and phylogenetic trees of COPD in LRE vs HRE (**a**) or PFT I vs PFT II (**c**). The relative color represented the more abundance bacterial taxonomy in each group. LEfSe analysis showed abundance of bacteria (LDA > 3) was altered as compared between (**b**) LRE and HRE; (**d**) PFT I and PFT II, respectively.

**
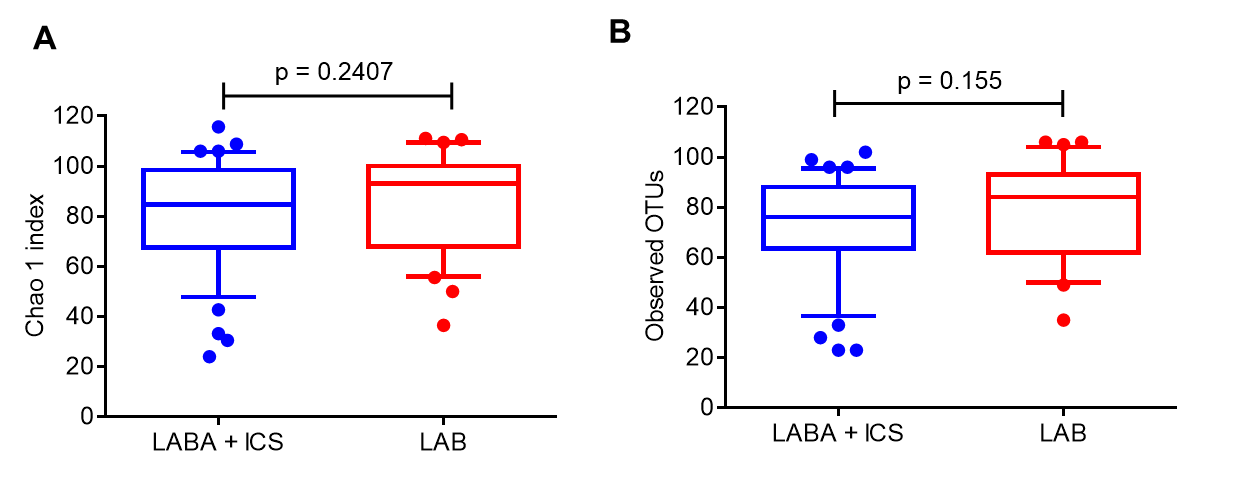
**

**Figure S3. Diversity index of sputum microbiome communities in stable COPD patients with different medications.**

The Chao1 index (a) and observed OTUs (b) are calculated in COPD patients with different medications. The box and whiskers plots shows the median, 10th and 90th percentile in each group. Bonferroni-adjusted p-values < 0.05/2 = 0.025 indicate significance.
